# Possible role of the α7 nicotinic receptors in mediating nicotine’s effect on developing lung – implications in unexplained human perinatal death

**DOI:** 10.1186/1471-2466-14-11

**Published:** 2014-02-01

**Authors:** Anna M Lavezzi, Melissa F Corna, Graziella Alfonsi, Luigi Matturri

**Affiliations:** 1 Department of Biomedical, Surgical and Dental Sciences, “Lino Rossi” Research Center for the study and prevention of unexpected perinatal death and SIDS, University of Milan, Via della Commenda 19, 20122 Milan, Italy

**Keywords:** Nicotinic receptors, Human lung development, Brainstem, SIDS, SIUDS, Nicotine, Maternal smoking in pregnancy

## Abstract

**Background:**

It is well known that maternal smoking during pregnancy is very harmful to the fetus. Prenatal nicotine absorption, in particular, is associated with alterations in lung development and functions at birth and with respiratory disorders in infancy. Many of the pulmonary disorders are mediated by the interaction of nicotine with the nicotinic receptors (nAChRs), above all with the α7 nAChR subunits that are widely expressed in the developing lung. To determine whether the lung hypoplasia frequently observed in victims of sudden fetal and neonatal death with a smoker mother may result from nicotine interacting with lung nicotinic receptors, we investigated by immunohistochemistry the possible presence of the α7 nAChR subunit overexpression in these pathologies.

**Methods:**

In lung histological sections from 45 subjects who died of sudden intrauterine unexplained death syndrome (SIUDS) and 15 subjects who died of sudden infant death syndrome (SIDS), we applied the radial alveolar count (RAC) to evaluate the degree of lung maturation, and the immunohistochemical technique for nAChRs, in particular for the α7 nAChR subunit identification. In the same cases, an in-depth study of the autonomic nervous system was performed to highlight possible developmental alterations of the main vital centers located in the brainstem.

**Results:**

We diagnosed a “lung hypoplasia”, on the basis of RAC values lower than the normal reference values, in 63% of SIUDS/SIDS cases and 8% of controls. In addition, we observed a significantly higher incidence of strong α7 nAChR immunostaining in lung epithelial cells and lung vessel walls in sudden fetal and infant death cases with a smoker mother than in age-matched controls. Hypoplasia of the raphe, the parafacial, the Kölliker-Fuse, the arcuate and the pre-Bötzinger nuclei was at the same time present in the brainstem of these victims.

**Conclusions:**

These findings demonstrate that when crossing the placenta, nicotine can interact with nicotinic receptors of both neuronal and non-neuronal cells, leading to lung and nervous system defective development, respectively. This work stresses the importance of implementing preventable measures to decrease the noxious potential of nicotine in pregnancy.

## Background

Maternal smoking during pregnancy has been associated with intrauterine growth retardation, low birth weight, neonatal morbidity and mortality, as well as an increased incidence of respiratory disorders and of an attention deficit in infants [1-6]. Nicotine, among thousands of compounds of tobacco smoke, is particularly involved in these pathological manifestations because, in cases of prenatal exposure, it readily crosses the placental barrier, so that levels of nicotine in the developing fetus closely mimic and also exceed the maternal levels [7].

Fetal damage caused by maternal smoking mainly result from the interaction of nicotine with nicotinic acetylcholine receptors (nAChRs) [8-10]. These receptors are ion channels in the cytoplasmic membrane consisting of a different combination of α and β subunits that can be opened not only by the neurotransmitter acetylcholine but also by nicotine – hence the name “nicotinic”. To date, a total of 10α and 4β different subunits have been described on the basis of the receptors sensitivity to nicotine [11].

It is well known that nAChRs regulate critical aspects of brain maturation during the intrauterine and early postnatal periods, mediating the neurochemical transmission of acetylcholine among the neurons [12-15]. Prenatal nicotine exposure significantly increases nAChR endogenous activation, above all in neuronal nuclei and/or in structures undergoing major phases of differentiation, that are consequently more sensitive to environmental stimuli.

The knowledge of the biology of nAChRs has expanded in recent years thanks to the identification of the nAChRs also in several peripheral organs, including the lungs [16,17]. Although the origin of lung damage caused by maternal smoking in pregnancy is likely multifactorial, many respiratory diseases may be mediated by the interaction of nicotine with the nicotinic receptors that are widely expressed in the airway cells of the developing lung [18]. In this scenario, the expression of nAChR provides a receptor-mediated mechanism that may help to understand, at the molecular level, how smoking affects lung development and leads to the well-documented decreased pulmonary function and increased respiratory symptoms observed in exposed infants after birth. Sekhon et al. [19,20], in experimental studies using specific antibodies for different nAChR subunits (*e.g.*, α3, α5, α7, β2), demonstrated a marked α7 immunostaining intensity, related to prenatal nicotine exposure, particularly in the airway epithelial cells and airway vessel walls of fetal and newborn lungs of monkeys. This means that nicotine absorption mainly upregulates the pulmonary expression of α7 nAChR, while other nicotinic receptor subtypes are more resistant. In addition, these authors indicated an association between α7 overexpression and an altered airway development, with a subsequent influence on respiratory health.

Previously, we have reported a high incidence of lung hypoplasia in subjects who died of Sudden Intrauterine Unexplained Death Syndrome (SIUDS) and Sudden Infant Death Syndrome (SIDS) [21,22]. In the present study we aimed to investigate whether the lung hypodevelopment frequently observed in these victims may be due, as shown in experimental studies, to a specific hyperactivation of the α7 nAChR subunit in cases of nicotine absorption in pregnancy.

## Methods

Among the many cases sent to our Research Center according to the guidelines stipulated by the Italian law n.31/2006 “*Regulations for Diagnostic Post Mortem Investigation in Victims of SIDS and Unexpected Fetal Death*”^a^ and diagnosed as SIUDS and/or SIDS, we selected 60 cases, precisely 45 sudden late fetal deaths (32–39 gestational weeks) and 15 sudden neonatal deaths (within the first month of life), provided with completeness of data, namely a detailed collection of clinical/environmental information, including the maternal smoking habit.

Twenty-seven mothers of the SIUDS/SIDS group (45%) claimed to smoke before and during pregnancy, while the remaining 32 (55%) denied they smoked.

We included in the study a control group consisting of 24 age-matched subjects (16 fetuses and 8 newborns) in which the autopsy established a precise cause of death (specifically, respiratory infections, cardiomyopathies, sepsis in infant deaths; cardiomyopathies and chorioamnionitis in fetal deaths). Five of the 24 mothers of the control group (21%) reported a smoking habit, while 19 mothers (79%) were non-smokers.

Table 1 summarizes the case profiles of the study.

**Table 1:** Case profiles of the study

	**Sudden perinatal deaths**		**Controls**	
	**SIUDS**	**SIDS**	**Fetuses**	**Newborns**
No. cases	45	15	16	8
Sex (M/F)	28/17	10/5	9/7	4/4
Age (range)	32-41 gw	2 h-27 d	33-40 gw	2 h-25 d
Maternal smoking	20	7	1	4

d = days; gw = gestational weeks; h = hours.

### Consent

Parents of all the victims of the study provided written informed consent to autopsy, with the Milan University “Lino Rossi” Research Center institutional review board approval.

A complete autopsy examination was carried out in every case including, in particular, an in-depth study of the brainstem and of the lungs. All organs were fixed in 10% phosphate-buffered formalin, processed and embedded in paraffin.

#### Brainstem examination

The brainstem was examined according to the protocol routinely applied in the “Lino Rossi” Research Center, available in our previous works [23,24] and in the web site http://users.unimi.it/centrolinorossi/en/guidelines.html.

The routine histological evaluation of the brainstem was focused on the main nuclei checking the vital functions: the locus coeruleus, the Kölliker-Fuse and the rostral raphe nuclei (magnum and caudal linear nuclei) in the rostral pons/caudal mesencephalon; the retrotrapezoid, the parafacial, the superior olivary and the median raphe nuclei in the caudal pons; the hypoglossus, the dorsal motor vagus, the tractus solitarius, the ambiguus, the pre-Bötzinger, the inferior olivary, the arcuate, the obscurus and the pallidus raphe nuclei in the medulla oblongata.

In addition we performed a morphometric analysis of the main nuclei with an Image-Pro Plus Image Analyzer (Media Cybernetics, Silver Spring, Maryland, USA) in serial histological sections. The following parameters were evaluated for every nucleus: neuronal density, (number of neurons per unit area-*mm*^*2*^) and volume. All the neurons with clearly defined edges and with a distinct nucleolus were counted using an optical microscope at 20× magnification. The volume was measured by three-dimensional reconstruction. A computer program developed by Voxblast (VayTek, Fairfield, Iowa) was used to digitize and display serial section reconstructions, to obtain volumetric measurements. The morphometric results were expressed as mean values and standard deviation. The statistical significance of direct comparisons between morphometric values obtained from sudden death cases and controls was determined using the analysis of variance. A diagnosis of hypoplasia related to a particular nucleus was formulated when its morphometric values were significantly lower than the reference values obtained from age-matched control cases.

#### Lung examination

Regarding the lung examination, the main focus of the study, samples were obtained from each lobe by cutting parallel to the frontal plane and passing through the hilus. The histological examination of the routinely stained sections by hematoxylin-eosin included the evaluation in terminal lung units (i.e., portions of parenchyma distal to the last respiratory bronchioles, identifiable by an incomplete epithelial lining) of the radial alveolar count (RAC). This is a reliable index of lung maturation in intrauterine and early postnatal development established by Emery and Mithal in 1960 [25], closely related to the gestational age in weeks for fetuses and to the postnatal age in months for newborns. The RAC is obtained by examining at least 10 random histological fields for each case in order to estimate the number of airspaces cut by a straight line drawn from the center of the most peripheral bronchiole to the nearest connective tissue septum or the pleura. Emery and Mithal also provided the RAC reference values at different ages. The normal mean value from 32 to 35 gestational weeks is 3.2 ± 0.9; from 36 to 39 gestational weeks 3.6 ± 0.9. The normal RAC from the first postnatal weeks to 4 months is 5.5 ± 1.4.

### nAChR immunohistochemistry

Immunohistochemical methods were applied to selected histological sections of lungs in order to evaluate the expression of α7 nAChRs by using specific rabbit polyclonal antibodies (aa 22–71, Abcam cod. ab10096). Sections, after dewax and rehydration, were immersed and boiled in a Citrate Buffer solution pH 6.0 (for α3) and in TRIS-EDTA Buffer (for α7 and β2) for the antigen retrieval with a microwave oven, having first blocked endogenous peroxidase by 3% hydrogen peroxide treatment. Then, sections were incubated with the primary antibodies overnight (diluitions: 1:75 for α3, 1:500 for α7 and 1:167 for β2) in a wet chamber. Samples were washed with PBS buffer and incubated with a secondary anti-rabbit antibody and then processed with a usual avidin-biotin-immunoperoxidase technique (VECTOR LABS, Burlingame, CA). Finally each section was counterstained with Mayer’s Hematoxylin and coverslipped.

A set of sections from each group of the study was used as negative control. Precisely, the tissue samples were stained using the same procedure but omitting the primary antibody in order to verify that the immunolabeling was not due to nonspecific labeling by the secondary antibody. In fact, if specific staining occurs in negative control tissues, immunohistochemical results should be considered invalid.

#### nAChR immunohistochemistry quantification

The degree of positive immunoreactivity in the lungs was defined for every case by two independent and blinded observers as the number of cells with strong unequivocal immunostaining, divided by the total number of consecutive cells counted in the epithelium surrounding both alveoli and bronchioles or divided by the total number of the counted cells in the parenchima, expressed as percentage (nAChR index: nAChR-I). nAChR-I was classified as: “Class 0” for no staining (negativity); “Class 1” when the index was < 10% (weak positivity); “Class 2” with a percentage of immunopositive cells between 10 and 30% (moderate positivity); “Class 3” with an index > 30% of the counted cells (strong positivity).

Comparison among the observations carried out by the two pathologists was performed employing Kappa statistics (Kappa Index- KI) to evaluate inter-observer reproducibility. The Landis and Koch [26] system of KI interpretation was used, where 0 to 0.2 is slight agreement, 0.21 to 0.40 indicates fair agreement, 0.41 to 0.60 is moderate agreement, 0.61 to 0.80 is strong or substantial agreement, and 0.81 to 1.00 indicates very strong or almost perfect agreement (a value of 1.0 being perfect agreement). This analysis revealed a very satisfactory Kappa Index (KI = 0.85).

### Markers for fibroblasts and collagen

– Immunostaining procedures for Fibroblast-specific protein-1 (FSP-1; 11 kD) of the S100 superfamily were applied to identify fibroblasts in the lung vessel walls. Skin samples were used as positive controls; negative controls were prepared omitting the primary antibody and replacing only with PBS during incubations. Sections from paraffin-embedded tissue blocks were treated using commercially supplied 1:400 diluted rabbit monoclonal antibodies FSP-1 (Millipore #07-2274). Slides were boiled for the antigen retrieval in 0.01 M citrate buffer (pH 6.0), using a microwave oven, at 600 W for 3 times at 5 min each, and finally cooled. A standard ABC technique avidin-biotin complex (Vectastain elite ABC KIT, PK-6101) was used with HRP-DAB to visualize and develop the antigen-antibody reaction. Sections were counterstained with Mayer’s hematoxylin, than coverslipped.

– Heidenhain’s trichrome staining (AZAN staining) was applied as histochemical staining procedure to identify the collagen around the airways and the vessels. In this method three acid dyes are used: azocarmine and a mixture of aniline blue and orange G. The azocarmine stain is combined with the mixture of aniline blue and orange G as counterstain, after mordanting with phosphotungstic acid. The collagen and cytoplasmic reticulum appear blue, muscle red to yellow, nuclear chromatine red.

### Statistical analysis

Histological and immunohistochemical data were tabulated and analyzed for differences comparing pairs of groups by using the analysis of variance (ANOVA). Statistical calculations were carried out with a SPSS statistical software (version 11.0; SPSS Inc., Chicago, IL, USA). The selected threshold level for statistical significance was p < 0.05.

## Results

### Lung examination in SIUDS/SIDS

#### Histology

**Figure 1:**
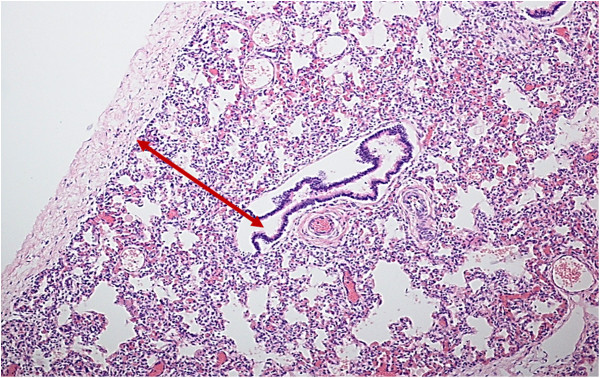
**Histological section of lung parenchima showing the radial alveolar count (RAC) method. SIDS case, 1 month-old** (RAC: 2.5; normal mean value for age: 5.5 ± 1.4). Hematoxylin-eosin stain; magnification: 10×.

In 38 cases (63%) of sudden death (33 SIUDS and 5 SIDS), a paucity of pulmonary alveoli was highlighted in histological sections demonstrating lower radial counts than the normal reference values (Figure 1). The mean RAC value was 2.1 ±0.4 in 12 fetuses aged 32–35 gws (normal value: 3.2 ± 0.9), and 2.7 ± 0.2 in 21 fetuses aged 36–39 gws (normal value: 3.6 ± 0.9), while the mean RAC value in the 5 newborns (aged from 1 to 3 postnatal months) was 3.0 ± 0.5 (normal value: 5.5 ± 1.4). These observations supported the diagnosis of “lung hypoplasia”.

#### nAChR expression

**Figure 2:**
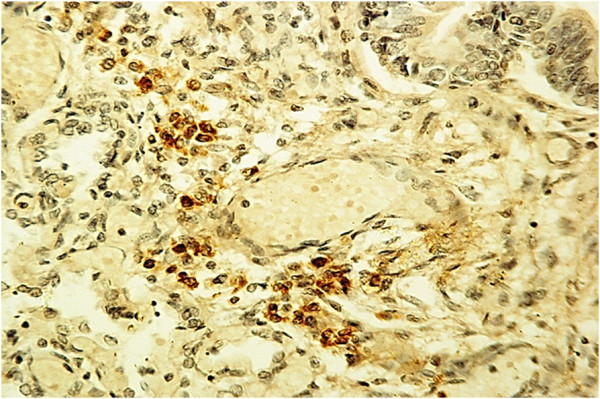
**Positive α7 nAChR expression in a group of cells of lung parenchyma – SIUDS case, 38 gestational weeks.** α7 nAChR immunostain; magnification: 20×.

**Figure 3:**
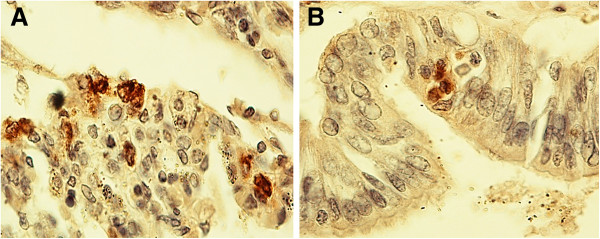
**Positive α7 nAChR epithelial cells surrounding in A) the lumen of an alveolus and in B) the lumen of a bronchiole – SIDS case, 2 month-old.** α7 nAChR immunostain; magnification: 40×.

**Figure 4:**
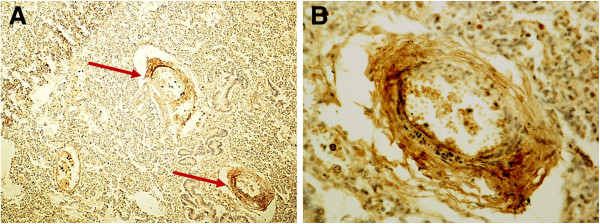
**Positive α7 nAChR in the wall of pulmonary vessels (see arrows).** One of the two vessels shown in **A)** is represented at higher magnification in **B) – SIUDS case, 38 gestational weeks.** α7 nAChR immunostain; magnification: **A)** 10×; **B)** 40×.

**Figure 5:**
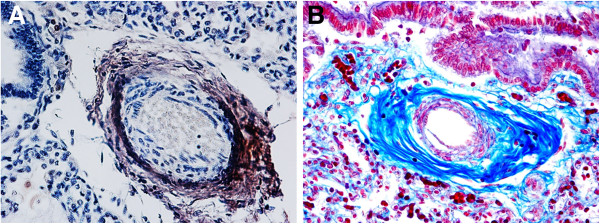
**Identification of pulmonary perivascular fibroblasts and collagen. A)** identification of fibroblastic cells around a lung vessel by FSP-1 immunohistochemistry (brown reaction product). **B)** Perivascular blue collagen fibers highlighted by histochemistry – SIDS case, 2 month-old. **A)** FSP-1 immunostain; magnification: 20x - **B)** AZAN stain; magnification 20×.

Examination of the immunostained preparations revealed a strong immunoreactivity for the nAChR α7 subunit (“Class 3” of nAChR-I) in densely packed cells of compact lung parenchyma areas (Figure 2) and in epithelium surrounding both alveoli and bronchioles (Figure 3) in 22 SIUDS and 8 SIDS victims. In these cases an intense α7 immunoreactivity was also frequently expressed in fibroblasts of the airway and vessel walls (Figure 4). The vascular walls, in particular, appeared thick due to the increase of connective tissue, as demonstrated by the intense histochemical positivity for the collagen and the positive immunostaining for fibroblasts (Figure 5).

### Lung examination in controls

#### Histology

Mostly, in control subjects the RAC was within the normal reference values. Nevertheless, a “low grade of lung hypoplasia” in 2 newborns (8%) who died of pneumonia in the first two months of life, with a slightly lower lung maturation index than the mean value for the age (RAC value: 4; mean reference value: 5.5 ± 1.4).

#### nAChR expression

A high α7 subunit immunoreactivity (“Class 3” of nAChR-I) was detected in the airway epithelium of 3 newborns, two of whom had died of respiratory infections and showed mild lung hypoplasia.

##### Correlation of lung observations with maternal smoking

A significant association between a strong expression of α7 nAChR, lung hypoplasia and prenatal cigarette smoke exposure (p < 0.01) occurred. In fact, 20 of the 27 cases with a smoker mother belonged to the SIUDS/SIDS group showing a high α7 immunopositivity and lower RAC values than the reference values. Moreover, the mothers of the 3 control infants with similar nAChR results and mild lung hypoplasia were active smokers in pregnancy.

### Brainstem examination in SIUDS/SIDS and controls

The in-depth histological examination, supported by the morphometric analysis, of the brainstem in SIUDS/SIDS cases frequently yielded a diagnosis of hypodevelopment of important nuclei and/or neuronal structures checking vital functions. Hypoplasia/agenesis of one or more nuclei of the raphe system (*i.e.*, obscurus, pallidus, median, magnum, caudal linear raphe nuclei), a complex of nuclei that plays a trophic role in brain development, being responsible for serotonergic transmission [27], was the most frequent alteration (observed in 28 SIUDS and 8 SIDS).

In 11 SIUDS with hypoplasia of the obscurus and pallidus raphe nuclei in the medulla oblongata, we observed an association with hypoplasia of the pontine parafacial and Kölliker-Fuse nuclei, consisting of “pre-inspiratory” neurons with the hierarchical function of starting the first breathing movements [28,29].

Hypoplasia of the medullary pre-Bötzinger nucleus, a critical structure for respiratory rhythmogenesis [30], and of the arcuate nucleus, involved in chemoreception [31], was simultaneously present in 12 SIUDS and 6 SIDS victims, five of whom showed agenesis of the raphe obscurus nucleus.

In most of these cases (15 SIUDS and 4 SIDS) the mother smoked during pregnancy.

A significant correlation was found between the neuropathological results and the above-reported lung alterations. In fact, in 20 SIUDS and 4 SIDS victims with a defective maturation of important brainstem centers, lung hypoplasia with a high α7 nicotinic receptor expression was discovered.

In controls hypoplasia of the arcuate nucleus was the only finding (observed in 3 cases), not related to maternal smoking. On the whole, SIUDS and SIDS cases displayed a significantly higher incidence of brainstem pathological findings as compared to age-matched controls (p < 0.01).

Table 2 summarizes the anatomopathological and immunohistochemical results obtained in this study.

**Table 2:** Pathological results

		**Sudden perinatal deaths**		**Controls**	
		**SIUDS**	**SIDS**	**Fetuses**	**Newborns**
		**n.45**	**n.15**	**n.16**	**n.8**
**Lung pathology**					
Lung hypoplasia		n.33 (73%)**	n.5 (33%)	n.0 (0%)	n.2 (25%)
***RAC*** (*mean value*)					
*Age*	*Reference value*		-	-	-
32-35 gw	3.2±0.9	2.1±0.4	-	-	-
36-39 gw	3.6±0.9	2.7±0.2	-	-	-
1-3 pm	5.5±1.4	-	3.0±0.5	-	4.0
***α7 nAChR strong immunopositivity***		n.22 (49%)**	n.8 (53%)*	n.0 (0%)	n.3 (37%)
**Brainstem pathology**					
Brainstem nuclei hypoplasia^(1)^		n.38 (84%)**	n.10 (67%)**	n.0 (0%)	n.3 (37%)
Raphe nuclei		28	8	-	-
Parafacial nucleus		11	8	-	-
Kölliker-Fuse nucleus		11	-	-	-
Pre-Bötzinger nucleus		12	6	-	-
Arcuate nucleus		12	6	-	3

gw= gestational weeks; pm= postnatal months; RAC= radial alveolar count.

^(1)^Individual victims may display any combination of brainstem alterations.

Statistical significant differences (SIUDS and SIDS *vs.* corresponding controls): *= p<0.05; **= p< 0.01.

Further research aimed at evaluating nicotinic receptor expression in the brainstem is now in progress in our laboratory, and will be the subject of a forthcoming publication.

## Discussion

The lung, during its long process of maturation from the embryonic phase of development in utero up to adolescence, is highly susceptible to damage caused by exposure to environmental toxicants, and particularly to tobacco smoke in pregnancy [32-34].

In pregnant smokers nicotine, that in amniotic fluid reaches similar or higher levels than those present in maternal plasma [7], when crossing the placenta can directly affect fetal lung development. This is facilitated by the fact that the metabolic activity of the fetal liver is not yet well developed so leading to a higher half-life of nicotine with the ability to interfere in the development of especially the most vulnerable organs, including the lung [35]. In addition the developing lung can be exposed during early childhood to nicotine ingestion through the breast milk from a smoking mother [36].

Previous experimental studies performed on Rhesus monkeys, an ideal model of pulmonary growth that is very similar to that of humans, demonstrated that nicotine administration during pregnancy causes lung hypodevelopment as a direct result of its interaction with the α7 subunit of the nicotinic receptors, that are widely distributed in the airways epithelium in prenatal life [19]. Similar results were more recently obtained by Wongtrakool et al. [37] in mouse. These authors established that prenatal nicotine exposure leads to a decrease in pulmonary function and in alveolar surface, through interactions with α7 nAChRs.

The involvement of this specific subunit in lung pathophysiology is also proven by its strong manifestation in tobacco-induced cancers. Nicotine contributes directly to pulmonary tumorigenesis through stimulation of the α7 nAChR subunits in target cells, as shown by their overexpression particularly in non-small cell lung carcinomas of patients who smoked [38,39].

Basing on these results, we propose a potential molecular mechanism responsible for the high incidence of lung hypoplasia in SIUDS and SIDS whose mothers smoked. We hypothesize that the overexpression of the α7 nicotinic receptors we found in these cases is the result of modifications of the corresponding gene expression. This is supported by the increased messenger RNA levels of several nAChR subunits (including α7 subunits) observed in the developing nervous system of rats as a consequence of nicotine absorption [40], leading to a remarkable increase in receptor protein synthesis. In human pulmonary cell cultures, Plummer et al. [41] likewise demonstrated a higher α7 mRNA and a consequent increase in α7 nicotinic receptor levels in the presence of nicotine-derived carcinogenic nitrosamine.

In our study, the α7 nAChR overexpression observed in lung epithelial cells in a high percentage of SIUDS/SIDS victims was exacerbated by the presence of developmental alterations of brainstem centers affecting not only breathing activity but, more in general, all the vital functions, thus amplifying the harmful effect of nicotine and leading to a fatal outcome. The results of the current research now in progress in our laboratory on the α7 nAChR expression in the brainstem, will validate this assertion. It is therefore clear that nicotine, after entering the fetal circulation and crossing the fetal blood–brain barrier, interacts with the α7 subunits and up-regulates its expression in both non-neuronal cells of the lung and in neuronal cells of the brainstem, thereby leading to impaired lung function and hypodevelopment of the respiratory neuronal centers.

The strong immunoexpression of the α7 nAChR subunit also observed in fibroblasts around lung vessels and airways must be underlined. The vascular walls, in particular, appear thicker, very likely due to interactions of nicotine with fibroblast receptors, thereby triggering a high collagen protein synthesis and hence causing accumulation of connective tissue. Nicotine can therefore directly stimulate α 7 nAChR-bearing fibroblasts to lay down an excessive amount of connective tissue.

These considerations are consistent with the report by Sekhon et al. [42] of a high expression of α7 subunits in fibroblasts of the lung vessels in monkey fetuses exposed to nicotine during pregnancy, with a significant accumulation of collagen in the tunica media.

Collagen deposition around the airways and pulmonary vessels certainly induces an increased airway resistance and decreased respiratory function, providing an explanation for the high incidence of persistent pulmonary hypertension observed in infants whose mothers smoked during pregnancy [43].

It is important to point out several limitations of this study. First, the exact mechanisms by which nicotine interacts with nAChR to alter lung development remain to be determined. This is due, in particular, to the impossibility to perform experimental studies in humans. However, based on our results we can hypothesize a link between α7 overexpression and delayed pulmonary development in SIUDS/SIDS victims. Secondly, pertaining to cigarette smoke exposure, is that available information on our cases did not provide evidence of an exposure ‘dose’. Furthermore, it should be considered that retrospective assessment of the maternal smoking, particularly if performed after a fatal event, is sometimes unreliable, due to the fact that mothers who smoke are reluctant to admit tobacco use, because of feelings of guilt [44]. Finally, it is important to point out that our study was focused on the role of the increased α7 receptor expression in developing lung in relation to nicotine absorption. Hovewer we detain that other nAChR subtypes could likely play important roles in mediating the effects of nicotine on lung development. Therefore we plan to extend the study, using a wider range of nAChRs.

## Conclusions

This article emphasizes the extreme vulnerability of the developing lung to maternal cigarette smoke absorption. In summary, we claim that nicotine in pregnant women is readily transported across the placenta, achieving sufficient levels to interact directly with the nicotinic receptors that are widely distributed in the lung cells in prenatal life. In particular, on the basis of our results and of previous experimental studies, we propose α7 nAChR as the main candidate mediating the pathobiologic effects of tobacco products on lung maturation and function. The specific interaction of nicotine molecules with α7 subunits, widespread in the cytoplasmic membrane of both lung epithelial cells and fibroblasts, leads to an overexpression of the corresponding receptors with a consequent severe inhibition of airway development *in utero*, as well as an airflow decrease in the first days of life and a high incidence of smoke-associated pediatric respiratory disorders. The relevance of this study is the significantly higher association highlighted among many factors (overexpression of α7 nAChRs, lung hypoplasia, developmental alterations of brainstem nuclei and maternal smoking) in SIUDS/SIDS, as compared to controls.

Despite the compelling evidence that prenatal nicotine absorption increases the incidence of pulmonary diseases in fetal and infant life, in addition to posing the well known risks of spontaneous abortion, preterm delivery, low birth weight and sudden perinatal death, a significant number of women continue to smoke during pregnancy.

The findings of this study further underline the importance of ensuring widespread information about the noxious potential of nicotine absorption in pregnancy, as well as the need to implement measures to prevent this major and avoidable cause of lung morbidity and mortality in fetuses and infants.

## Endnote

^a^This law decrees that all infants suspected of SIDS, suddenly died in Italian regions within the first year of age, as well as all fetuses who died without any apparent cause (SIUDS), must undergo an in-depth anatomo-pathological examination, particularly of the autonomic nervous system.

## Competing interests

The authors declare that they have no competing interests.

## Authors’ contributions

AML planned the study, analyzed the data and wrote the manuscript with collaborative input and extensive discussion with LM. MFC and GA carried out the immunohistochemical and the histochemical study and participated in the evaluation of the results. All authors read and approved the final manuscript.

## Pre-publication history

The pre-publication history for this paper can be accessed here:

http://www.biomedcentral.com/1471-2466/14/11/prepub
